# Detection of emphysema progression in alpha 1-antitrypsin deficiency using CT densitometry; Methodological advances

**DOI:** 10.1186/1465-9921-9-21

**Published:** 2008-02-13

**Authors:** David G Parr, Martin Sevenoaks, ChunQin Deng, Berend C Stoel, Robert A Stockley

**Affiliations:** 1 Department of Respiratory Medicine, University Hospitals of Coventry and Warwickshire, Clifford Bridge Road, Coventry, CV2 2DX, UK; 2 Lung Investigation Unit, University Hospital of Birmingham, Edgbaston, Birmingham, B15 2TH, UK; 3 Talecris Biotherapeutics, Research Triangle Park, NC 27709, USA; 4 Division of Image Processing, Department of Radiology, Leiden University Medical Centre, Leiden 2300-RC, The Netherlands

## Abstract

**Background:**

Computer tomography (CT) densitometry is a potential tool for detecting the progression of emphysema but the optimum methodology is uncertain. The level of inspiration affects reproducibility but the ability to adjust for this variable is facilitated by whole lung scanning methods. However, emphysema is frequently localised to sub-regions of the lung and targeted densitometric sampling may be more informative than whole lung assessment.

**Methods:**

Emphysema progression over a 2-year interval was assessed in 71 patients (alpha 1-antitrypsin deficiency with PiZ phenotype) with CT densitometry, using the 15^th ^percentile point (Perc15) and voxel index (VI) -950 Hounsfield Units (HU) and -910 HU (VI -950 and -910) on whole lung, limited single slices, and apical, central and basal thirds. The relationship between whole lung densitometric progression (ΔCT) and change in CT-derived lung volume (ΔCT_Vol_) was characterised, and adjustment for lung volume using statistical modelling was evaluated.

**Results:**

CT densitometric progression was statistically significant for all methods. ΔCT correlated with ΔCT_Vol _and linear regression indicated that nearly one half of lung density loss was secondary to apparent hyperinflation. The most accurate measure was obtained using a random coefficient model to adjust for lung volume and the greatest progression was detected by targeted sampling of the middle third of the lung.

**Conclusion:**

Progressive hyperinflation may contribute significantly to loss of lung density, but volume effects and absolute tissue loss can be identified by statistical modelling. Targeted sampling of the middle lung region using Perc15 appears to be the most robust measure of emphysema progression.

## Background

Emphysema is defined as 'abnormal, permanent enlargement of airspaces distal to the terminal bronchioles, accompanied by destruction of their walls, and without obvious fibrosis' [1]. The proteolytic tissue destruction that is pathognomonic of emphysema should directly cause a reduction in lung density, but additional loss arises from lung hyperinflation that is secondary to increased lung compliance. Lung density changes can be measured using computed tomography (CT) scanning, and CT lung densitometry is now widely accepted to be the most sensitive and specific measure of emphysema *in vivo *[2-7]. However, several technical issues remain unresolved. The level of inspiration during scan acquisition influences lung density and, in sequential studies, variability in inspiratory level will reduce the reproducibility of longitudinal data. Consequently, a number of methods have been proposed that either control lung volume during scan acquisition [8-10] or adjust density measurements to correct for the influence of volume effects [2,3,10-12]. These latter methods require an assessment of lung volume derived from CT imaging acquired using a whole lung volumetric scanning protocol and will negate any density change that is secondary to hyperinflation.

Although whole lung imaging has additional advantages, for example, comprehensive assessment of emphysema severity and distribution, emphysema is not evenly distributed throughout the lung, but is located in characteristic regions [13]. Disease progression may occur by the extension of emphysema in a predictable pattern and, therefore, targeted sampling from within a whole lung imaging series may identify disease progression (and response to emphysema-modifying therapy) with greater discrimination than whole lung densitometric assessment.

We hypothesised that the progression of CT densitometry would relate to changes in lung volume, including progressive hyperinflation and, therefore, the influence of inspiratory level could be predicted and controlled by statistical modelling. In addition, it was hypothesised that disease progression would occur by the extension of emphysema from basal and/or apical regions and, therefore, the greatest densitometric change would be detected in the middle regions of the lung.

## Methods

### Subjects

Subjects with severe alpha 1-antitrypsin deficiency (AATD) with a PiZ phenotype who had been selected from those attending our centre for a previous study [13] were invited to attend after an interval of 2 years for further assessment. Ethics approval was given by the local research ethics committee, and all subjects gave written informed consent. The alpha 1-antitrypsin concentration and phenotype were confirmed as described previously [14] and, at the time of assessment, all subjects were in the stable clinical state and none had received alpha 1-antitrypsin augmentation therapy. All patients gave written informed consent. The study was approved by relevant local ethics review committees and was conducted in accordance with the Declaration of Helsinki and Good Clinical Practice guidelines.

### Lung function testing

Lung function testing was performed at baseline according to the British Thoracic Society/Association of Respiratory Technicians and Physiologists (BTS/ARTP) guidelines, as described previously [14,15], and results expressed as a percentage of predicted values [16].

### Computed tomography

**Figure 1:**
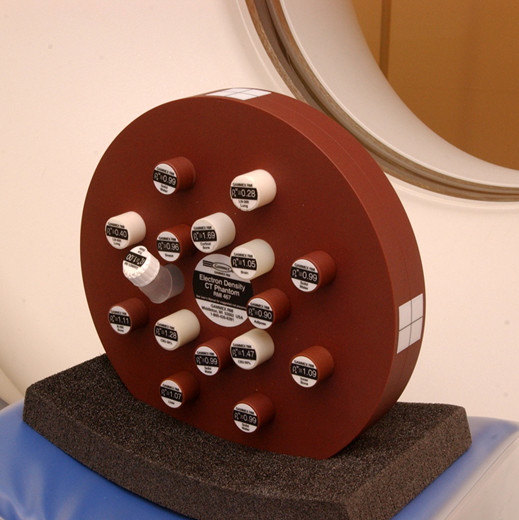
Electron density phantom. Three electron density rods (LN300, LN450 and 'solid water') were removed and located over the sternum during scan acquisition for use in internal quality assurance.

Patients were scanned in the supine position (with shoulder abduction), at full inspiration, using a 'volume' protocol on a General Electric *Lightspeed *scanner in the helical mode and without the use of intravascular contrast, as previously described [13]. CT calibration included daily automatic air calibration, as advised by the manufacturer (General Electric Medical Systems, Milwaukee, WI, USA). Additional quality assurance data was obtained using 3 electron density component rods from an RMI467 electron density CT phantom (Gammex – RMI Ltd, Nottingham, UK) (Figure 1). Two rods with density values equivalent to lung tissue (LN300, LN450), and one rod with equivalent density to water ('solid water'), were positioned over the mid-sternum during scan acquisition (see 'CT densitometry', below). Imaging was performed at baseline and repeated after of an interval of approximately 2 years.

### CT densitometry

**Figure 2:**
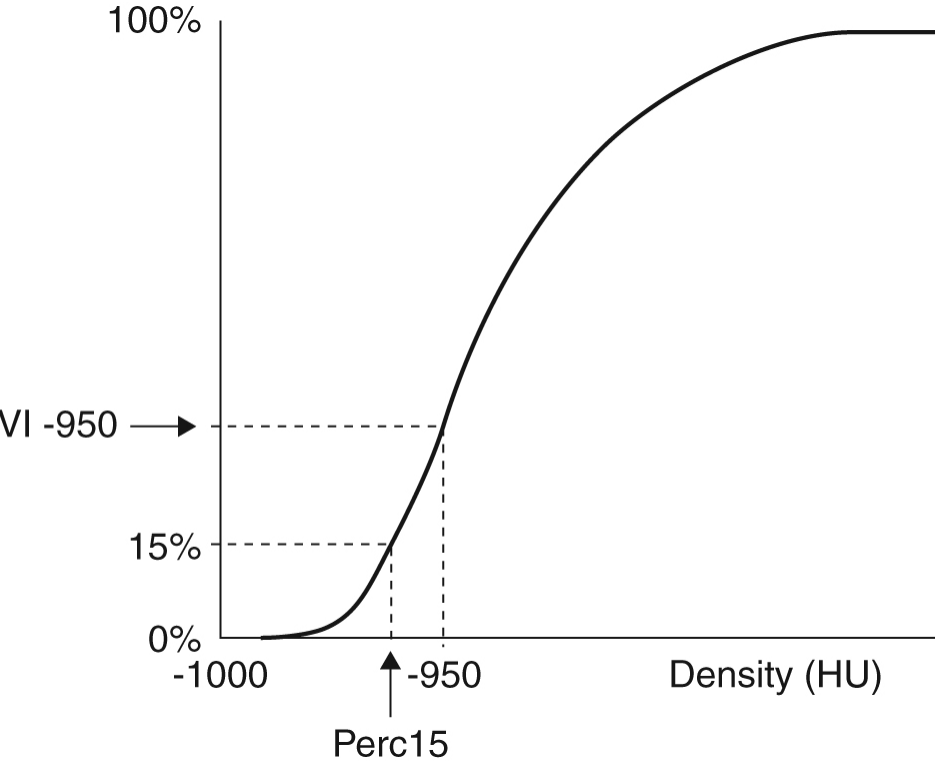
**Cumulative voxel distribution histogram showing derivation of voxel index and percentile point parameters**. Voxel index (VI) below 950 Hounsfield Units (-950HU) is defined as the proportion of lung voxels of low density below a threshold of -950HU and increases with worsening emphysema. The 15^th ^percentile point (Perc15) is defined as the cut-off value in HU below which 15% of all voxels are distributed and, as a true measure of density, this parameter consequently decreases with worsening emphysema.

Voxel index at a threshold of -950 Hounsfield Units (VI-950HU) and -910 HU (VI-910HU) and the 15^th ^percentile point (Perc15) (Figure 2) were measured for whole lung and additional single images selected from the whole lung series, representing the upper (level of the aortic arch) and lower (level of the inferior pulmonary veins) zones using computer software (Pulmo-CMS, Medis Specials, Leiden, the Netherlands) as described previously [13]. In addition, the lung was divided into 3 regions (apical, middle and basal) using the sequential axial image numbers. When possible, an equal number of image slices was allocated to each region, but when the total number was not exactly divisible by 3, the additional slices were allocated to the basal third. Densitometric parameters were calculated on these three regions as described above.

Adjustment of all densitometric parameters was performed using an internal air calibration method, as previously described [6], and additional quality assurance was obtained by densitometric assessment of each electron density rod using the Pulmo-CMS 'region of interest' (ROI) facility. The whole lung volume that was achieved with a full inspiratory manoeuvre during scan acquisition (CT_Vol_) was calculated using Pulmo-CMS, as previously described [13].

### Relationship between densitometric progression and lung volume change

Densitometric progression (ΔCT) and lung volume difference (ΔCT_Vol_) were calculated by measuring the difference between baseline and follow-up measurements for each parameter from a whole lung series and the annual rate of change was estimated using time interval as the denominator.

### Statistical analysis

Data were analysed using the Statistical Analysis System (SAS) version 9.1.3, (SAS Institute, Cary, USA). Associations between ΔCT and ΔCT_Vol _were assessed by Pearson's correlation coefficient.

CT densitometric progression was assessed using 3 approaches; (1) the differences between the baseline and follow-up values were assessed with a paired *t*-test, coefficient of variation (CV%) and relative standard deviation (RSD%); (2) adjustment of densitometric parameters for inspiratory volume variability by linear regression of ΔCT versus ΔCT_Vol _using an estimation of the intercept ΔCT_Vol _= 0; (3) a random coefficients model using densitometric outcome as the dependent variable, time (years) as the fixed effect, CT volume as longitudinal covariate, and intercept and time (years) as random effects. The volume-adjusted progression in densitometry was estimated from the slope (coefficient for time variable).

## Results

### Baseline characteristics

Seventy-one patients agreed to participate in the follow-up study. Fifty-five (78%) patients were index cases (defined as individuals diagnosed with AAT deficiency following presentation with lung disease) and 37 (52%) were male. Thirty-eight (54%) patients had previously smoked and 8 (11%) patients continued to smoke. The baseline physiological characteristics expressed as the mean ± standard deviation of percent predicted values are as follows; FEV_1 _57.1 ± 27.1, vital capacity 106.2 ± 23.1, residual volume 126.5 ± 36.0, total lung capacity (TLC) (helium dilution; TLC_He_) 115.3 ± 13.7, diffusing capacity for carbon monoxide (TlCO) 64.0 ± 19.3 and transfer coefficient (KCO) 65.2 ± 20.6.

### CT calibration

The mean interval between scans was 2.03 ± 0.44 years. CT calibration was maintained over the course of the study as indicated by the calibration data in Table 1. Internal air calibration data was recorded for all scans (n = 71), but electron density rods were utilised in 32 patients.

**Table 1:** CT calibration data

	Baseline	Follow-up	Change from baseline
Internal air calibration (n = 68)	-998.7 ± 0.9	-997.5 ± 1.9	1.2 ± 5.5
LN300 electron density rod (n = 32)	-717.5 ± 2.4	-716.4 ± 3.2	1.1 ± 3.0
LN450 electron density rod (n = 32)	-552.4 ± 2.6	-551.6 ± 2.8	0.8 ± 3.4
'Solid' water electron density rod (n = 32)	-7.8 ± 3.5	-9.7 ± 3.6	1.9 ± 2.7

All values are presented as the mean ± standard deviation in Hounsfield Units (HU).

### Relationship between TLC and inspiratory volume measured from CT

**Figure 3:**
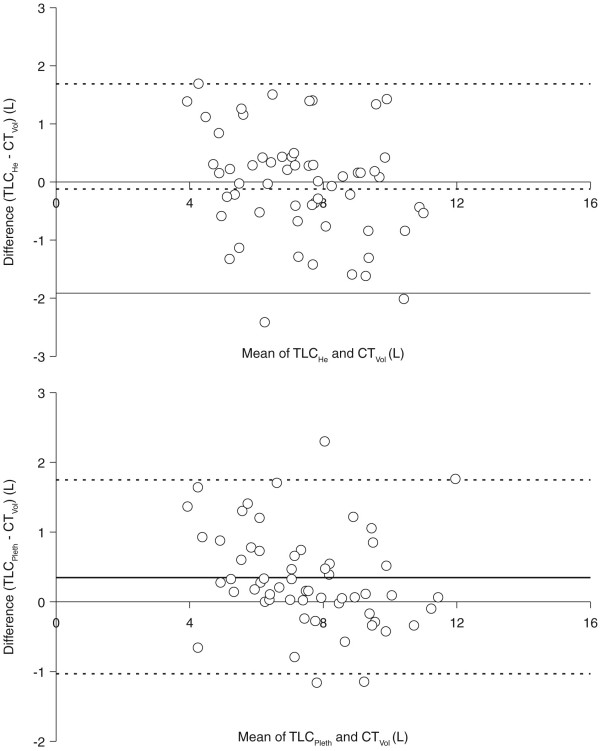
**Bland-Altman plots indicating difference between (A) total lung capacity measured by helium dilution (TLC_He_) and inspiratory lung volume achieved during scan acquisition (CT_Vol_), and (B) total lung capacity measured by body plethysmography (TLC_Pleth_) and CT_Vol_**. Continuous line represents mean difference and dashed lines represent mean difference +/- 2 standard deviations.

Fifty-eight patients had TLC assessments performed using both body plethysmography (TLC_pleth_) and TLC_He _methods, and the correlation between these measures was good (r = 0.907, p < 0.001). The correlation between CT_Vol _and TLC_pleth _(r = 0.938, p < 0.001) was better than the correlation between CT_Vol _and TLC_He _(r = 0.889, p < 0.001). Bland-Altman plots [17] indicated that CT_Vol _systematically under-estimated in comparison to TLC_pleth _but was similar to TLC_He _(see Figures 3A and 3B).

### Relationship between densitometric progression and lung volume change

**Figure 4:**
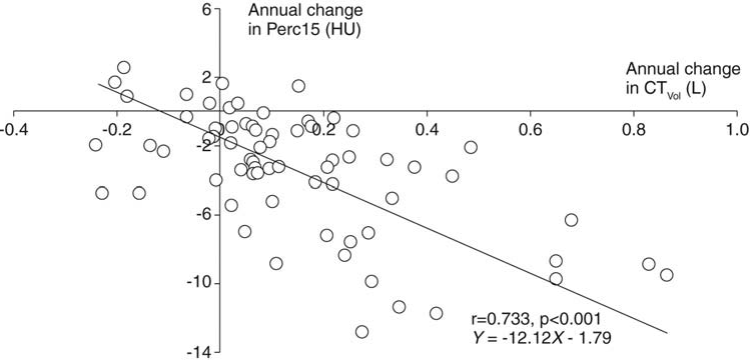
**Correlation and regression of annual change in CT_Vol _with annual change in Perc15**.

There was a close correlation between the rate of change in lung volume measured from CT imaging (ΔCT_Vol_) and the rate of densitometric progression assessed from whole lung sampling, using Perc15 (r = -0.733, p < 0.001) (Figure 4), VI-950 (r = 0.600, p < 0.001) and VI-910 (r = 0.719, p < 0.001).

### Progression of CT densitometry

#### 'Raw' densitometric progression

*S*tatistically significant densitometric progression was identified using endpoint analysis with all densitometric parameters (Table 2).

**Table 2:** Densitometric progression ('raw' data)

Mean ± SD	Baseline	Follow-up	Change from baseline	t	p *value*	Annual change
WL Perc15 (HU)	-939.09 ± 33.44	-946.16 ± 29.26	-7.06 ± 8.97	-6.64	< 0.0001	-3.53
WL VI-950 (%)	15.08 ± 10.63	17.75 ± 11.35	2.67 ± 3.34	6.74	< 0.0001	1.34
WL VI-910 (%)	36.29 ± 18.28	40.48 ± 18.06	4.18 ± 5.14	6.86	< 0.0001	2.09

WL, whole lung; Perc15,15^th ^percentile point (measured in Hounsfield Units); SD, standard deviation; VI-950, voxel index at a threshold of -950HU (measured in %); VI-910, voxel index at a threshold of -910HU (measured in %); annual change, change in outcome measure without adjustment for lung volume.

#### Adjustment for lung volume using linear regression

The regression equations for each densitometric parameter, shown in Table 3, demonstrate that the measured change in lung density was closely associated with changes in lung volume. The intercept (ΔCT at ΔCT_Vol _= 0) indicates the change in lung density that was not due to change in inspiratory level during scan acquisition and was, for each densitometric parameter, equivalent to approximately 50% of the mean change in lung density (Table 2). The gradient of the slope was greatest for Perc15 (12.12), and greater for VI-910 (6.58) than for VI-950 (2.54). When standardised for the change from baseline for each densitometric parameter (Table 2) (slope/annual change from baseline), the influence of inspiratory level on densitometric progression was greatest for Perc15 (3.43), and greater for VI-910 (3.14) than for VI-950 (1.89). Correcting for differences in lung volume reduced the magnitude of densitometric progression, but the changes remained highly statistically significant for all densitometric parameters (Table 3).

**Table 3:** Densitometric progression adjusted for lung volume using linear regression

Variable	Linear regression models	Intercept, mean ± SE (95% CI)	t	p value	Annual change
WL Perc15	ΔPCP = -12.12*ΔCT_Vol _-3.57	-3.57 ± 0.83 (-5.48, -1.65)	-3.72	0.0004	-1.79
WL VI-950	ΔVI-950 = 2.54* ΔCT_Vol _+ 1.94	1.94 ± 0.44 (1.07, 2.81)	4.45	< 0.0001	0.97
WL VI-910	ΔVI-910 = 6.58* ΔCT_Vol _+ 2.28	2.28 ± 0.57 (1.15, 3.42)	4.01	0.0002	1.14

WL, whole lung; Perc15, 15^th ^percentile point (measured in Hounsfield Units); VI-950, voxel index at a threshold of -950HU (measured in %); VI-910, voxel index at a threshold of -910HU (measured in %); Δ, change over study period; SE, standard error; 95% CI, 95% confidence interval; annual change, change outcome measure from baseline to follow-up incorporating adjustment for lung volume, estimated from the intercept (ΔCT_Vol _= 0).

#### Adjustment for lung volume using a random coefficient model

Perc15 was the most sensitive measure of densitometric progression after adjusting for lung volume variability, and selective sampling of the middle third was the most robust method for detecting change, based on the *t *value (Table 4). The influence of lung volume accounted for 32.09% of the measured loss in lung density when assessed using VI-950, compared with 42.21% of the progression using Perc15 and 44.5% of the progression using VI-910.

**Table 4:** Densitometric progression adjusted for lung volume using random coefficient model

Variable	Annual change, mean ± SE (95% CI)	t	p value for annual rate	p value for volume
Whole lung analysis				
WL Perc15	-2.13 ± 0.44 (-3.01, -1.24)	-4.82	< 0.0001	< 0.0001
WL VI-950	0.90 ± 0.19 (0.52, 1.29)	4.72	< 0.0001	< 0.0001
WL VI-910	1.16 ± 0.25 (0.66, 1.65)	4.67	< 0.0001	< 0.0001

Single slice analysis				
UZPerc15	-2.02 ± 0.53 (-3.08, -0.97)	-3.82	0.0003	< 0.0001
UZ VI-950	0.60 ± 0.21 (0.18, 1.02)	2.84	0.0057	< 0.0001
UZ VI-910	0.94 ± 0.33 (0.29, 1.59)	2.88	0.0051	< 0.0001
LZ Perc15	-1.93 ± 0.58 (-3.07, -0.78)	-3.34	0.0013	< 0.0001
LZ VI-950	1.02 ± 0.32 (0.39, 1.65)	3.22	0.0019	< 0.0001
LZ VI-910	1.34 ± 0.38 (0.58, 2.1)	3.52	0.0007	< 0.0001

Targeted sampling				
UT Perc15	-2.54 ± 0.62 (-3.77, -1.32)	4.13	< .0001	< 0.0001
UT VI-950	0.67 ± 0.22 (0.24, 1.10)	3.11	0.0027	< 0.0001
UT VI-910	1.28 ± 0.44 (0.42, 2.15)	2.95	0.0042	< 0.0001
MT Perc15	-2.94 ± 0.49 (-3.91, -1.97)	-6.04	< 0.0001	< 0.0001
MT VI-950	1.20 ± 0.24 (0.73, 1.67)	5.08	< 0.0001	< 0.0001
MT VI-910	1.78 ± 0.35 (1.08, 2.47)	5.12	< 0.0001	< 0.0001
LT Perc15	2.85 ± 0.61 (-4.07, -1.64)	-4.68	< 0.0001	< 0.0001
LT VI-950	1.28 ± 0.26 (0.76, 1.80)	4.96	< 0.0001	< 0.0001
LT VI-910	1.57 ± 0.29 (0.99, 2.15)	5.37	< 0.0001	< 0.0001

WL, whole lung; UZ, upper zone single slice; LZ, lower zone single slice; ATl, apical third; MT, middle third; LT, lower third; SE, standard error; Perc15, 15^th ^percentile point (measured in Hounsfield Units); VI-950, voxel index at a threshold of -950HU (measured in %); VI-910, voxel index at a threshold of -910HU (measured in %). The random coefficient model consists of outcome measurement as the dependent variable, time (year) as the fixed effect, volume as a time-dependent covariate, intercept and time (year) as random effects. The slope (coefficient for time variable) from the model is the estimated annual change adjusted for volume.

## Conclusion

The current study shows that emphysema progression can be detected over a 2-year period by CT densitometry using several methods for image analysis. Highly statistically significant progression was demonstrated utilising both percentile point and voxel index parameters. Densitometric progression was closely related to changes in lung volume and a significant proportion of the density loss appeared to be related to apparent 'progressive hyperinflation'. The incorporation of statistical methods to adjust for differences in inspiratory level between scans indicated that, although increasing lung volume accounted for some of the loss of lung density, statistically significant changes could still be demonstrated following elimination of this component of the signal. It is logical to conclude that the remaining changes are likely to reflect absolute change in lung mass and this is of fundamental interest. There has been debate concerning whether loss of tissue mass occurs in emphysema. The proteinase/anti-proteinase theory predicts that loss of lung elastin is central to the pathophysiological process [18,19]. However, animal experiments showed that the initial loss was rapidly followed by elastin re-synthesis as the emphysema developed [20]. Furthermore, fibrosis is often present in emphysematous lung [20-22], which would increase lung density. Our data indicate that part of the reduction in lung density as emphysema progresses is related to a net loss of tissue. Consequently, the inclusion of our statistical methods in future studies will enable differential assessment of these 2 principal components of densitometric progression. In particular, this method of analysis will be of importance in the characterisation of treatment effect in therapeutic trials of potential disease-modifying therapy.

Other approaches that have been proposed to reduce the variability arising from inspiratory level have been applied to individual patient data, either by controlling inspiration during scan acquisition [9], or by adjusting lung density to a chosen lung volume [2,3,12]. Whilst these methods may reduce the variability of longitudinal densitometry, thereby improving the statistical power of interventional studies, they remain contentious. In contrast, the method utilised in the current study employs a valid statistical approach that is recognised and accepted for the comparison of grouped data in randomized, placebo-controlled trials. Furthermore, the application of this method to group data enables differential assessment of density change that arises from net tissue loss and progressive hyperinflation, and this may be pertinent in trials of potential emphysema modifying therapy. Notwithstanding this additional advantage, it is recognised that the current method cannot be utilised to correct individual patient data and, therefore, the aforementioned alternative methods of volume correction are likely to remain of potential use.

The magnitude of difference in CT_Vol _that was apparent in our cohort is surprising, and much greater than would be expected from the hyperinflation that is secondary to increased compliance associated with emphysema progression. It is possible that some of the increase in CT_Vol _reflects either a patient learning effect, due to familiarisation with the required inspiratory manoeuvres on repeat imaging, or from changes in the coaching methods employed by the radiography staff. A component of the measured increase in lung volume will undoubtedly reflect emphysema-related hyperinflation and, although it is desirable that this signal is not eliminated, it was not possible to retain this component using the methodology that was employed. Nevertheless, the data at baseline indicate that CT_Vol _was closely related to physiologically-derived TLC measurements and, therefore, it would be possible in a long-term study during which emphysema-related hyperinflation might be expected to be of greater significance, that CT densitometric parameters could be adjusted to a given lung volume derived from progressive changes in TLC measured in the physiology laboratory. Unfortunately, the current study did not include repeat measures of TLC in all patients and further studies are therefore needed to explore this potential method.

Contemporary scanning protocols for densitometric assessment of emphysema commonly acquire volumetric data and encompass the whole lung, but emphysema is frequently localised within characteristic regions of the lung [13], particularly in the early stages of disease. Consequently, densitometric assessment of the whole lung may be superfluous and more sensitive detection of emphysema progression may be achieved by targeted sampling. This is suggested from previous studies that have identified differential rates of progression between densitometric assessment of single slices in the upper and lower lung regions [4]. The natural history of disease progression is likely to involve progressive extension from the initial sites of emphysema development. In AATD, this will most commonly occur in a basal to apical direction but in usual chronic obstructive pulmonary disease (COPD) in an apical to basal direction. There is no longitudinal data of sufficient duration to confirm this premise, but these patterns of emphysema extension may explain why mortality is best predicted by upper zone densitometric indices in subjects with AATD [23] and by lower zone indices in subjects with usual COPD [24]. Our group has previously shown that approximately one third of subjects with AATD have an 'atypical' distribution of emphysema that includes greater involvement of the apical regions [13]. Consequently, we hypothesised that, in an unselected group of subjects with AATD, targeted sampling of the middle lung region would be the most sensitive method for assessing disease progression, as this would detect extension of both basal and apical emphysema. The results verify this hypothesis, and suggest that in future studies of potential emphysema-modifying therapy, targeted sampling may be of greater discriminative value in identifying a treatment effect that retards progression than whole lung assessment. Notwithstanding this potential advantage, highly statistically significant differences in lung density were demonstrated for all sampling methods and for all of the densitometric parameters that were utilised. The Perc15 method was the most sensitive parameter, and these data support previous comparative studies [2,7] and the recommendations of a working party [5]. However, the relationship between ΔCT and ΔCT_Vol _suggests that there is a greater influence of inspiratory level on Perc15 than VI-950, and the use of volume control or adjustment is likely to be more critical when Perc15 is used for emphysema monitoring studies.

CT calibration has been shown to influence CT lung densitometry [6,25,26] and internal calibration methods have indicated scanner inconsistency over time, despite the application of routine calibration practice. The current study utilised a previously validated method of internal calibration [6] and, in addition, explored the use of electron density rods for further quality assurance. Quality assurance data using air densitometry acquired from patient images indicated that there was a gradual change in scanner performance over the course of the study (Table 1). Densitometric data derived from ROI measurements of the electron density rods indicated that the drift in air calibration was not an isolated artefact and that the magnitude of change was similar across a wide density spectrum (Table 1). The likely effect of these changes would be a small reduction in the apparent rate of emphysema progression but correction was achieved using a previously validated internal calibration method [6]. Additional internal calibration data from the electron density rods indicated that the methodological assumptions of this approach were valid; in particular, the change in air densitometric values obtained from patient images could be used to assess and, therefore, adjust the densitometric value of tissue with density intermediate between that of water and air, including the lung.

In conclusion, we have shown that CT densitometry is a statistically robust tool for monitoring emphysema progression and that appropriate contemporary scanning techniques are reproducible for use in longitudinal studies. Lung density change is greatly influenced by variation in inspiratory level, but the accuracy of lung densitometry is improved by the incorporation of statistical modelling to adjust for the effects of lung volume. Perc15 is the most sensitive index for monitoring progression and additional sensitivity is achieved by densitometric assessment of the middle region of the lung. Targeted sampling may, therefore, be more sensitive than whole lung assessment for the identification of treatment effect in CT densitometric studies of potential emphysema-modifying therapy.

## Competing interests

Dr Parr's and Dr Sevenoaks' salaries were paid for by a non-commercial grant from Bayer plc and Dr Parr acts as a consultant for Talecris Biopharmaceuticals and Hoffman La Roche. Dr Stoel is consultant for Hoffman La Roche, Talecris Biopharmaceuticals, CSL Behring and Bioimaging Technologies Inc. Professor Stockley has lectured widely for non-promotional purposes to several pharmaceutical companies (GlaxoSmithKline, Bayer and Eli Lilly) and acts on advisory boards with an interest in COPD (Astra Zeneca, GlaxoSmithKline, Talecris Biopharmaceuticals, Schering-Plough and Baxter Pharmaceuticals) and as a consultant (Etiologics). In addition, significant non-commercial research grants have been awarded by Astra Zeneca and Bayer.

## Authors' contributions

Every author has contributed to reviewing the paper. DGP and MS performed the image analysis. DGP and CD performed the statistical analysis. DGP drafted the manuscript. BCS developed the software used for image analysis (Pulmo-CMS). RAS is the principal investigator of the project, obtained funding of and supervised the project. All authors read and approved the final manuscript.
